# Deep learning-based automated detection and multiclass classification of focal interictal epileptiform discharges in scalp electroencephalograms

**DOI:** 10.1038/s41598-023-33906-5

**Published:** 2023-04-25

**Authors:** Yoon Gi Chung, Woo-Jin Lee, Sung Min Na, Hunmin Kim, Hee Hwang, Chang-Ho Yun, Ki Joong Kim

**Affiliations:** 1 Division of Pediatric Neurology, Department of Pediatrics, Seoul National University Bundang Hospital, Seoul National University College of Medicine, Seongnam-si, Republic of Korea; 2 Department of Neurology, Seoul National University Bundang Hospital, Seoul National University College of Medicine, Seongnam-si, Republic of Korea; 3 Kakao Healthcare, Seongnam-si, Republic of Korea; 4 Department of Pediatrics, Seoul National University Children’s Hospital, Seoul National University College of Medicine, Seoul, Republic of Korea

**Keywords:** Neurology, Engineering

## Abstract

Detection and spatial distribution analyses of interictal epileptiform discharges (IEDs) are important for diagnosing, classifying, and treating focal epilepsy. This study proposes deep learning-based models to detect focal IEDs in electroencephalography (EEG) recordings of the frontal, temporal, and occipital scalp regions. This study included 38 patients with frontal (*n* = 15), temporal (*n* = 13), and occipital (*n* = 10) IEDs and 232 controls without IEDs from a single tertiary center. All the EEG recordings were segmented into 1.5-s epochs and fed into 1- or 2-dimensional convolutional neural networks to construct binary classification models to detect IEDs in each focal region and multiclass classification models to categorize IEDs into frontal, temporal, and occipital regions. The binary classification models exhibited accuracies of 79.3–86.4%, 93.3–94.2%, and 95.5–97.2% for frontal, temporal, and occipital IEDs, respectively. The three- and four-class models exhibited accuracies of 87.0–88.7% and 74.6–74.9%, respectively, with temporal, occipital, and non-IEDs F1-scores of 89.9–92.3%, 84.9–90.6%, and 84.3–86.0%; and 86.6–86.7%, 86.8–87.2%, and 67.8–69.2% for the three- and four-class (frontal, 50.3–58.2%) models, respectively. The deep learning-based models could help enhance EEG interpretation. Although they performed well, the resolution of region-specific focal IED misinterpretations and further model improvement are needed.

## Introduction

Interictal epileptiform discharges (IEDs) are electroencephalography (EEG) biomarkers of epilepsy important for diagnosing, classifying, and monitoring the disease and for selecting anti-seizure medication^1–4^. In current practice, IEDs are manually detected during EEG interpretation. This process is highly labor-intensive and time-consuming because it depends on visual interpretation by neurology specialists^5–7^. Ongoing investigations have endeavored to develop an automated technique that could efficiently detect IEDs in EEG recordings at an acceptable accuracy^8^. Recently, deep learning techniques have been widely accepted as the main strategy for building automated IED detectors for scalp^9–17^ and intracranial^18–21^ EEG recordings.

Epilepsy, a chronic disorder of the brain that causes recurrent spontaneous seizures, is categorized as focal or generalized. Recurrent seizures originating within a neuronal network limited to one hemisphere, unifocal or multifocal, are core features of focal epilepsy. Analyzing the spatial distribution of IEDs in focal epilepsy is of fundamental importance to properly classify it and determine the cortical generators of the epileptic activity. Nevertheless, the irritative zone might not exactly match the epileptogenic zone^22,23^. Most deep learning-based investigations performed binary classification of scalp EEG recordings into IED and non-IED, regardless of their location. Studies have reported automated detectors for centrotemporal IEDs, a characteristic EEG marker for self-limited epilepsy with centrotemporal spikes^13,16,24^. One study reported an automated detector trained by frontal, temporal, parietal, and occipital IEDs in patients with focal epilepsy, although they did not attempt to classify the IEDs according to their spatial properties^12^. Deep-learning-based models to automatically detect and classify multiple region-specific IEDs have not yet been reported.

Despite continuous improvements, limited diagnostic performance and accuracy have been major obstacles to deep learning-based EEG analysis, preventing its clinical application. EEG artifacts and normal EEG variants are major sources of false IED identification. Furthermore, the IED types vary substantially by the location in the brain. To improve the diagnostic performance and extend the application range, a deep learning-based IED detector should be trained and validated to detect IEDs in each region. The ideal algorithm should be able to detect and localize the IEDs.

This study aimed to develop a deep learning-based model to detect focal IEDs in the frontal, temporal, and occipital regions of scalp EEG recordings. Binary models classified each focal region as IED and non-IED. Subsequently, multiclass classification models categorized the location into frontal, temporal, occipital, or non-IEDs.

## Methods

### Dataset

This study retrospectively analyzed scalp EEG recordings of 38 patients diagnosed with focal epilepsy (15 females, 23 males; mean age 20.1 ± 16.2 years) and 232 controls with paroxysmal non-epileptic neurological events such as syncope, headache, or vertigo, but without seizures, epilepsy, or any other neurological disease (132 females, 100 males; mean age 12.9 ± 3.1 years) from the pediatric and adult neurology clinics of Seoul National University Bundang Hospital. Patients with focal epilepsy were categorized into frontal (*n* = 15; three females, 12 males; age 11.4 ± 3.6 years), temporal (*n* = 13; seven females, six males; mean age 35.3 ± 20.2 years), and occipital (*n* = 10; five females, five males; mean age 13.2 ± 2.7 years) IED groups. The Institutional Review Board of Seoul National University Bundang Hospital approved this study (No. B-2106-688-105) and waived the requirement for informed consent due to the retrospective nature of the study. This study was conducted following the principles of the Declaration of Helsinki.

The EEG recordings were obtained in awake-resting and sleeping states using 32-channel digital EEG systems (Grass Telefactor Inc., West Warwick, RI, USA). EEG was recorded for at least 30 min at a sampling frequency of 200 Hz, with a notch filter of 60 Hz and 19 electrodes, following the international 10–20 system. Chloral hydrate (50 mg/kg, maximum 1000 mg) was used as a sedative for pediatric patients if clinically indicated. EEG recordings were re-referenced to the average reference montages with 19 channels and band-pass filtered between 1 and 70 Hz for further analysis. Two epileptologists (HH and HK) reviewed the EEG data of the 38 patients with focal epilepsy to annotate IEDs. Each IED was defined from the beginning of its spike, sharp wave, or spike/sharp-wave complex to the end of its discharge component. The epileptologists also confirmed that no IED was present in the EEG recordings of the 232 control individuals.

### IED annotation

A total of 4557 IEDs (2112 frontal, 1176 temporal, and 1269 occipital) were annotated from the 38 patients with focal epilepsy. The number of IEDs per EEG recording was 141 ± 129, 90 ±  ± 99, and 127 ± 68 in the frontal, temporal, and occipital regions, respectively. The mean lengths of frontal, temporal, and occipital IEDs were 0.45, 0.52, and 0.48 s, respectively. Detailed information on the patients with focal IEDs is presented in Table 1.

**Table 1:** Profiles of patients with frontal, temporal, and occipital IEDs.

Frontal							Temporal							Occipital						
Subject No	Age (yr)	Sex	Duration of IEDs			Number of IEDs	Subject No	Age (yr)	Sex	Duration of IEDs			Number of IEDs	Subject No	Age (yr)	Sex	Duration of IEDs			Number of IEDs
			Max (s)	Min (s)	Avg (s)					Max (s)	Min (s)	Avg (s)					Max (s)	Min (s)	Avg (s)	
1	9	M	0.70	0.25	0.43	134	1	17	F	0.83	0.30	0.52	189	1	14	F	0.69	0.19	0.33	86
2	8	M	0.58	0.33	0.48	6	2	11	M	0.65	0.30	0.42	65	2	13	F	0.70	0.24	0.44	90
3	12	M	0.86	0.20	0.45	549	3	7	M	0.72	0.26	0.43	15	3	9	F	1.03	0.26	0.45	281
4	16	M	0.77	0.28	0.48	113	4	18	M	0.56	0.25	0.34	20	4	16	F	0.75	0.25	0.42	167
5	12	M	0.48	0.44	0.45	4	5	63	M	0.76	0.22	0.44	345	5	17	F	0.86	0.28	0.52	159
6	7	F	0.80	0.22	0.49	147	6	39	M	0.68	0.22	0.44	108	6	12	M	0.81	0.31	0.50	158
7	10	M	0.61	0.17	0.35	69	7	31	M	0.83	0.29	0.49	210	7	11	M	1.05	0.31	0.61	127
8	11	M	0.67	0.22	0.41	107	8	63	F	0.86	0.36	0.65	39	8	10	M	0.91	0.38	0.51	57
9	13	M	0.76	0.33	0.53	149	9	51	F	0.59	0.28	0.46	40	9	16	M	0.76	0.33	0.53	89
10	12	F	0.45	0.22	0.30	39	10	63	F	0.46	0.30	0.39	11	10	14	M	0.76	0.39	0.51	55
11	12	M	0.77	0.24	0.48	227	11	45	F	2.60	0.31	1.01	52							
12	18	F	0.69	0.24	0.46	103	12	25	F	1.27	0.31	0.58	50							
13	17	M	0.75	0.27	0.43	117	13	26	F	0.86	0.36	0.61	32							
14	7	M	0.92	0.27	0.49	167														
15	7	M	0.84	0.27	0.47	181														
Sum						2112							1176							1269
Average					0.45	141						0.52	90						0.48	127

*IED* interictal epileptiform discharge, *M* male, *F* female.

### Classification models

Binary classification models were constructed individually for the frontal, temporal, and occipital IEDs. Multiclass classification models were constructed to distinguish focal IEDs from other IEDs in various regions. One-dimensional (1D) and two-dimensional (2D) convolutional neural networks (CNNs) were adopted for the classification models, with multichannel EEG time series as input data. EEG recordings with IEDs were segmented into 1.5 s epochs that contained the IEDs at their center and spanned − 0.75 s to + 0.75 s from the IED center (hereafter referred to as focal IED epochs). The EEG recordings of the controls were segmented into 1.5 s epochs at random time points (hereafter referred to as non-IED epochs). At here, an epoch denoted a 1.5 s EEG segment.

**Figure 1:**
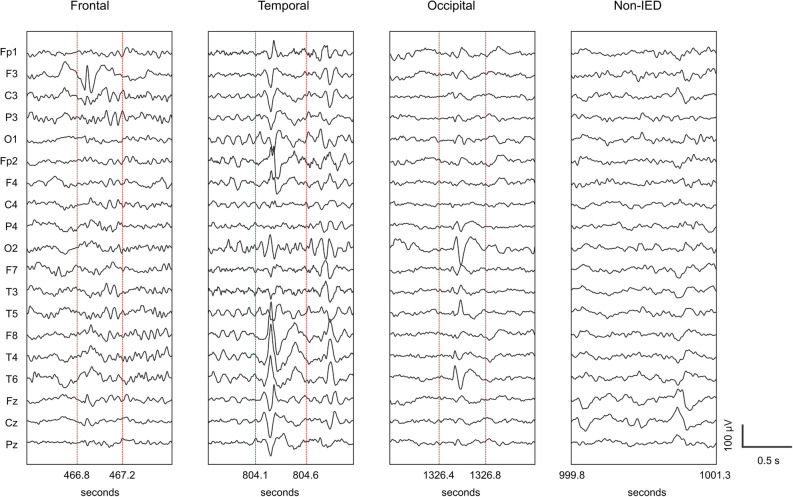
Representative epochs corresponding to focal interictal epileptiform discharges (IEDs) and non-IED.

The focal IED epochs were split randomly into training, validation, and test sets at a ratio of 6:2:2. Frontal, temporal, and occipital IED epochs were handled separately in the individual binary classification to classify IED and non-IED epochs. A set of focal IED epochs was handled collectively in the multiclass classification models to classify frontal, temporal, occipital IED, and non-IED epochs. The focal IED epochs were augmented by random jittering between − 50 ms and + 50 ms from the center of each epoch to handle imbalanced data distributions caused by much smaller number of focal IED epochs compared to non-IED epochs. The non-IED epochs were randomly under-sampled to match the number of augmented focal IED epochs at a 1:1 ratio. We made the focal IED epochs have fully shaped IEDs to ensure a sufficient number of clean-labeled training data for the robustness of our deep learning-based classification^25^. Representative focal IED and non-IED epoch images are shown in Fig. 1.

The binary classification models’ performance was assessed using sensitivity, specificity, accuracy, and the area under the receiver operating characteristic curve (AUC). The multiclass classification models’ performance was assessed using precision and recall, and the F1-score was calculated. We used Python 3.8 with Tensorflow 2.2, compute unified device architecture 10.1, four NVIDIA TITAN V graphic cards with 12 GB memory to implement classification models; and sklearn.metrics module for the performance evaluation.

### CNN architecture

We adopted a CNN architecture consisting of three convolution layers, batch normalization, and max pooling layers. Multi-channel EEG time series were fed into the first convolution layer with an input size of 19 × 300 (number of channels × number of data points). Fully connected layers had output sizes of two (IED and non-IED epochs), three (temporal and occipital IED and non-IED epochs), and four (frontal, temporal, and occipital IED and non-IED epochs) in accordance with classification types. For our 1D CNN-based classification, the three convolution layers had 64, 128, and 64 filters, with a kernel size of 19 × 6 and a stride of 1. For our 2D CNN-based classification, the three convolution layers had 32, 64, and 32 filters with a kernel size of 1 × 6 and a stride of 1. We set the kernel size considering the minimum size of spikes and waves each described in a previous study on EEG characteristics of IEDs^26^. 1D or 2D max pooling layers were applied to the three convolution layers with a pooling size of 3, 2, and 1 in order and a stride of 2. Batch normalization was applied to each convolution layer to provide regularization and enhance training speed. Categorical cross-entropy and Softmax were used as the loss and activation functions, respectively, for the binary and multiclass classifications. The root mean square propagation (RMSprop)^27^ was used as an optimizer with a learning rate of 1 × 10^−5^. The model with the highest validation accuracy during training was selected for testing by the model checkpoint in Keras to avoid overfitting. The total number of training epochs was 300 with a batch size of 64.

### Feature visualization

We applied t-distributed stochastic neighbor embedding (t-SNE) for 2D visualizations of frontal, temporal, occipital, and non-IED epoch features. t-SNE is a statistical tool for dimensionality reduction that minimizes the mismatch between the joint probabilities of high- and low-dimensional data points^28^. We extracted the focal IED and non-IED epoch features from the flattened layers in the 1D and 2D CNN-based classification models. We used sklearn.manifold.TSNE module in the scikit-learn library with default parameters that included a component number of 2, perplexity of 30, and early exaggeration of 12. No additional statistical analyses were performed using the t-SNE results.

### Patient-level evaluation

We additionally performed leave-one-patient-out cross-validation for patient-level evaluation of the multiclass classification of focal IEDs. We excluded frontal IEDs to avoid potential effects of eye-related artifacts on the classification. Therefore, we performed the leave-one-patient-out cross-validation with respect to the three-class classification of temporal, occipital, and non-IEDs using the EEG recordings of 13 and 10 patients with temporal and occipital IEDs, respectively. We trained 2D CNN-based three-class classification models using the focal IED epochs of *N*-1 patients, where *N* was the total number of patients with temporal or occipital IEDs. Then, we evaluated performance of the classification models using the focal IED epochs of the remaining one patient. The performance of the patient-level evaluation was assessed using a detection rate defined as the number of correctly identified model-classified focal IED epochs divided by the total number of focal IED epochs for individual patients. All the focal IED epochs in this procedure were augmented by random jittering as described above.

## Results

### Binary classification

**Figure 2:**
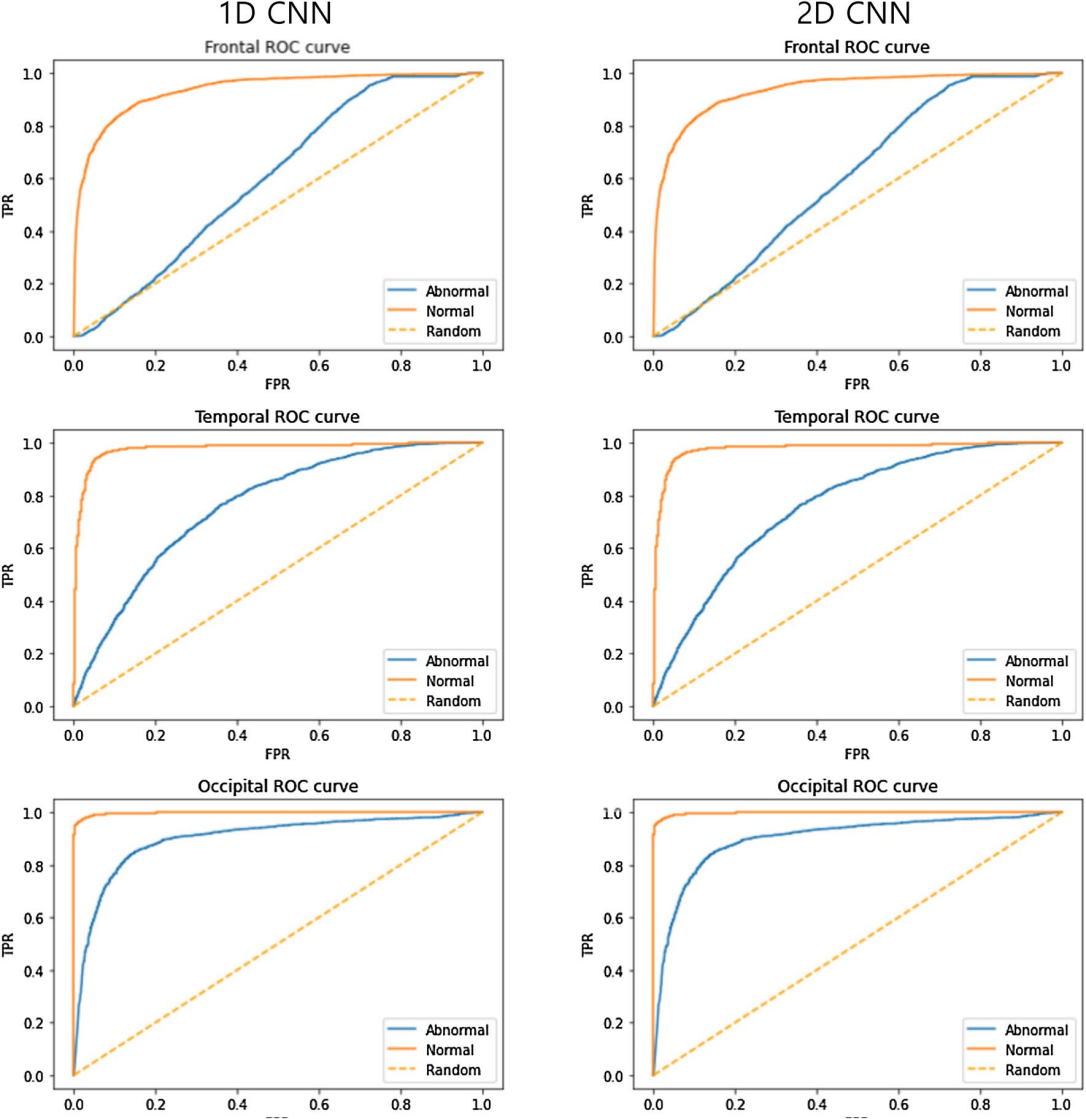
Binary classification performance with receiver operating characteristic (ROC) curves for frontal, temporal, and occipital IEDs. The upper, middle, and lower panels in each column represent the ROC curves of the frontal, temporal, and occipital IEDs, respectively. Abnormal and Normal in each small box indicate the performance using the non-IED epochs from patients and controls, respectively. CNN, convolutional neural network; IED, interictal epileptiform discharge; TPR, true positive rate; FPR, false positive rate.

The numbers of epochs in the individual binary classification for the frontal, temporal, and occipital IEDs were 38,946, 27,952, and 24,222, respectively. The 1D CNN-based binary classification exhibited accuracies of 86.4% (sensitivity, 86.5%; specificity, 86.3%), 94.2% (95.3% and 93.1%), and 97.2% (98.5% and 95.8%) for frontal, temporal, and occipital IEDs, respectively. The 2D CNN-based binary classification exhibited respective accuracies of 79.3% (85.3% and 73.4%), 93.3% (96.4% and 90.2%), and 95.5% (96.6% and 94.4%). The accuracies for frontal IEDs were 7.8% and 10.7% lower than those for temporal and occipital IEDs, respectively, in the 1D CNN-based classification and 14.0% and 16.2%, respectively, in the 2D CNN-based classification. The frontal IED accuracy in the 2D CNN-based classification was 7.1% lower than in the 1D CNN-based classification (Table 2). The respective AUCs for the frontal, temporal, and occipital IEDs were 93.7%, 97.9%, and 99.8% for the 1D CNN-based classification and 87.2%, 98.0%, and 98.6% for the 2D CNN-based classification (Normal in Fig. 2).

**Table 2:** Diagnostic performance of the binary classification models.

	1D CNN-based classification				2D CNN-based classification			
	Sensitivity (%)	Specificity (%)	Accuracy (%)	AUC (%)	Sensitivity (%)	Specificity (%)	Accuracy (%)	AUC (%)
Frontal	86.5	86.3	86.4	93.7	85.3	73.4	79.3	87.2
Temporal	95.3	93.1	94.2	97.9	96.4	90.2	93.3	98.0
Occipital	98.5	95.8	97.2	99.8	96.6	94.4	95.5	98.6

*CNN* convolutional neural network, *AUC* area under the receiver operating characteristic curve.

To explore the effect of the source of non-IED epochs on the binary classification, we examined additional performance using the non-IED epochs from the patients with focal epilepsy. In this case, the non-IED epochs were selected at random time points outside the focal IED epochs in the EEG recordings of the patients. The respective AUCs for the frontal, temporal, and occipital IEDs were 60.3%, 76.2%, and 90.1% for the 1D CNN-based classification and 63.7%, 72.9%, and 78.1% for the 2D CNN-based classification (Abnormal in Fig. 2), which were noticeably lower than the above.

### Three-class classification

**Figure 3:**
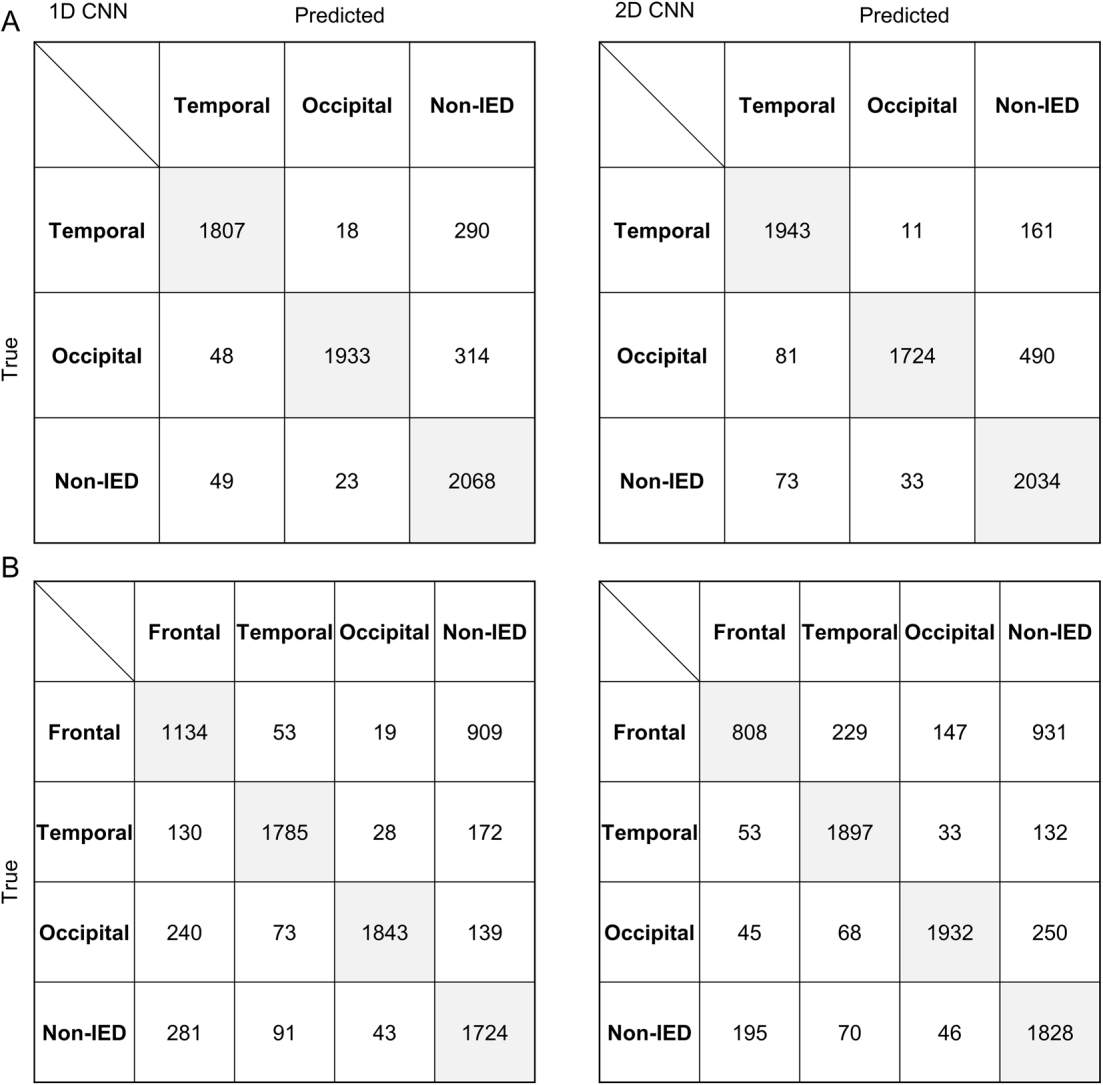
Confusion matrices for the 1D and 2D CNN-based multiclass classification results with the number of correctly and incorrectly classified focal and non-IEDs. Performance of the three-class (upper panels) and four-class (lower panels) classifications. CNN, convolutional neural network; IED, interictal epileptiform discharge.

The three-class classification (excluding the frontal IEDs) included 32,709 epochs with a near 1:1:1 ratio between the temporal (11,439), occipital (10,575), and non-IED (10,695) epochs. The 1D CNN-based three-class classification exhibited F1 scores of 89.9% (precision, 94.9%; recall, 85.4%), 90.6% (97.9% and 84.2%) and 86.0% (77.4% and 96.6%) for temporal, occipital, and non-IEDs, respectively, with an overall accuracy of 88.7%. The 2D CNN-based three-class classification showed respective F1 scores of 92.3% (92.7% and 91.9%), 84.9% (97.5% and 75.1%), and 84.3% (75.8% and 95.1%), with an overall accuracy of 87.0% (Table 3). The precision for non-IEDs was 17.5% and 20.5% lower than that for the temporal and occipital IEDs, respectively, in the 1D CNN-based classification, and 16.9% and 21.8%, respectively, in the 2D CNN-based classification. The number of temporal and occipital IEDs misclassified as non-IEDs (false negative focal IEDs) was 290 and 314, respectively, in the 1D CNN-based classification and 161 and 490, respectively, in the 2D CNN-based classification (Fig. 3a). The 2D CNN-based classification exhibited a 6.4% higher recall for temporal IED but a 9.1% lower recall for occipital IED than the 1D CNN-based classification.

**Table 3:** Diagnostic performance of the multiclass classification models.

	1D CNN-based classification			2D CNN-based classification		
	Precision (%)	Recall (%)	F1 Score (%)	Precision (%)	Recall (%)	F1 Score (%)
Three class						
Temporal	94.9	85.4	89.9	92.7	91.9	92.3
Occipital	97.9	84.2	90.6	97.5	75.1	84.9
Non-IED	77.4	96.6	86.0	75.8	95.1	84.3
Four class						
Frontal	63.5	53.6	58.2	73.4	38.2	50.3
Temporal	89.2	84.4	86.7	83.8	89.7	86.6
Occipital	95.3	80.3	87.2	89.5	84.2	86.8
Non-IED	58.6	80.6	67.8	58.2	85.5	69.2

*CNN* convolutional neural network, *IED* interictal epileptiform discharge.

### Four-class classification

The four-class classification included 43,269 epochs with a near 1:1:1:1 ratio among the frontal (10,560), temporal (11,439), occipital (10,575), and non-IED (10,695) epochs. The 1D CNN-based four-class classification exhibited F1 scores of 58.2% (precision, 63.5%; recall, 53.6%), 86.7% (89.2% and 84.4%), 87.2% (95.3% and 80.3%), and 67.8% (58.6% and 80.6%) for frontal, temporal, occipital, and non-IEDs, respectively, with an overall accuracy of 74.9%. The 2D CNN-based four-class classification showed respective F1 scores of 50.3% (73.4% and 38.2%), 86.6% (83.8% and 89.7%), 86.8% (89.5% and 84.2%), and 69.2% (58.2% and 85.5%), with an overall accuracy of 74.6% (Table 3).

The precision for non-IEDs was 5.0%, 30.6%, and 36.8% lower than for the frontal, temporal, and occipital IEDs, respectively, in the 1D CNN-based classification, and 15.2%, 25.6%, and 31.3%, respectively, in the 2D CNN-based classification. The numbers of temporal, occipital, and non-IEDs misclassified as frontal IEDs (false positives for frontal IED) were 130, 240, and 281, respectively, in the 1D CNN-based classification and 53, 45, and 195, respectively, in the 2D CNN-based classification. The numbers of frontal, temporal, and occipital IEDs misclassified as non-IEDs (false negative focal IEDs) were 909, 172, and 139, respectively, in the 1D CNN-based classification, and 931, 132, and 250, respectively, in the 2D CNN-based classification (Fig. 3b).

**Figure 4:**
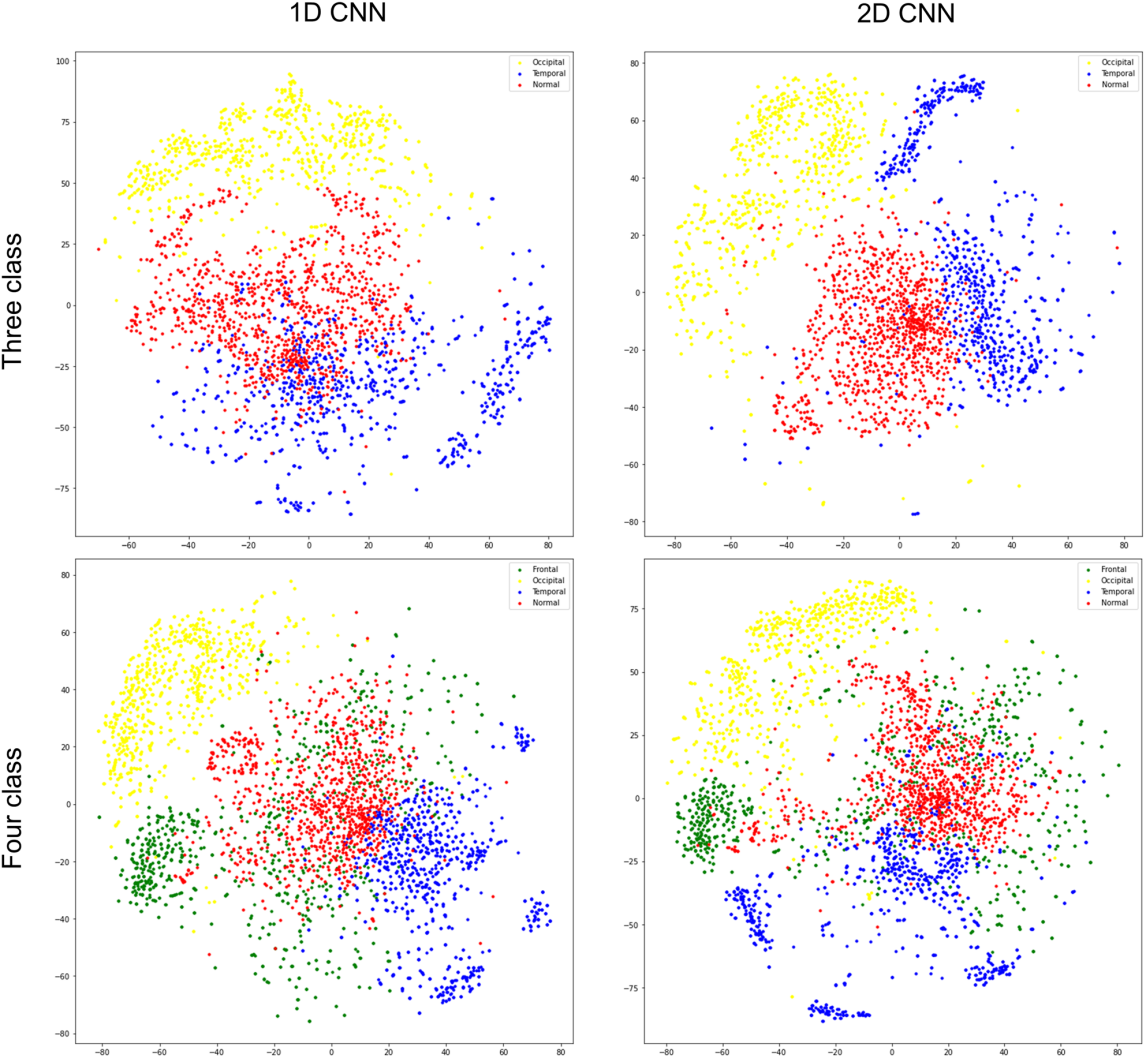
2-dimensional feature visualization for frontal, temporal, occipital, and non-IEDs using t-SNE. Feature visualization for the three-class (upper panels) and four-class (lower panels) classification. Green, blue, yellow, and red dots represent frontal, temporal, occipital, and non-IEDs, respectively. Owing to the large number of epochs (32,709 in the three-class classification and 43,269 in the four-class classification), we randomly selected 1000 of each class for visualization (3000 in the three-class classification and 4000 in the four-class classification). IED: interictal discharge and t-SNE: t-distributed stochastic neighbor embedding.

The 2D visualization of the frontal, temporal, occipital, and non-IED features is shown in Fig. 4. We qualitatively examined that those features were apparently separated from each other, in particular for the 2D CNN-based three-class classification. We could observe large overlaps between frontal and non-IED features for the four-class classification.

### Patient-level evaluation

Our patient-level evaluation for the 2D CNN-based three-class classification showed a mean detection rate of 82.8 ± 22.6% and 87.6 ± 15.2% for temporal and occipital IEDs, respectively, averaged over each corresponding number of patients. For the temporal IED detection, 13 patients had detection rates from 34.3% to 100% (> 90% in seven patients, < 66% in two patients, where 33% was a chance for each class). For the occipital IED detection, 10 patients had detection rates from 49.0 to 100% (> 90% in six patients, < 66% in one patient). Detailed results for the patient-level evaluation are shown in Table 4.

**Table 4:** Performance of patient-level evaluation using leave-one-patient-out cross-validation for 2D CNN-based three-class classification.

Subject No	Age (yr)	Sex	Number of IEDs	Total number of focal IED epochs	Number of model-classified focal IED epochs			Detection rate (%)
					Temporal	Occipital	Non-IEDs	
Patients with temporal IEDs								
1	17	F	189	1890	1827	9	54	96.7
2	11	M	65	650	223	184	243	34.3
3	7	M	15	150	61	32	57	40.7
4	18	M	20	200	194	0	6	97.0
5	63	M	345	3440	2394	6	1,040	69.6
6	39	M	108	1080	943	1	136	87.3
7	31	M	210	2100	1867	209	24	88.9
8	63	F	39	390	390	0	0	100.0
9	51	F	40	400	363	26	11	90.8
10	63	F	11	110	110	0	0	100.0
11	45	F	52	520	520	0	0	100.0
12	25	F	50	500	358	142	0	71.6
13	26	F	32	320	320	0	0	100.0
Mean ± SD								82.8 ± 22.6
Patients with occipital IEDs								
1	14	F	86	860	46	421	393	49.0
2	13	F	90	900	43	726	131	80.7
3	9	F	281	2810	12	2661	137	94.7
4	16	F	167	1670	172	1411	87	84.5
5	17	F	159	1600	89	1448	63	90.5
6	12	M	158	1580	0	1576	4	99.7
7	11	M	127	1280	0	1280	0	100.0
8	10	M	57	570	7	563	0	98.8
9	16	M	89	890	56	834	0	93.7
10	14	M	55	550	0	465	85	84.5
Mean ± SD								87.6 ± 15.2

*CNN* convolutional neural network, *IED* interictal epileptiform discharge, *SD* standard deviation.

## Discussion

This study implemented deep learning-based automated binary focal IED detectors with accuracies of 79.3–86.4%, 93.3–94.2%, and 95.5–97.2% for frontal, temporal, and occipital IEDs, respectively, and multiclass focal IED detectors with accuracies of 87.0–88.7% and 74.6–74.9% for three and four (frontal IED included) IED classes, respectively, on scalp EEG recordings. Frontal IEDs were associated with a low detection performance, probably due to eye-related artifacts in the frontal region. The inclusion of spatial information by applying 2D CNN to multi-channel scalp EEG recordings provided mixed effects on the focal IED detection performance.

The major finding of the current study was that the implemented individual binary IED detectors showed a significant discrepancy in their focal IED detection performance among the three brain regions. Detection of temporal and occipital IEDs exhibited accuracies of 94.2 and 97.2%, respectively, while detection of frontal IEDs showed inferior performance with an accuracy of 86.4%.

Previous studies have reported binary classification performance with sensitivities of 47.4–99.0%, specificities of 79–98.0%, and AUCs of 0.935–0.980^10–12,14,15,17^, regardless of the IED location. Assessment of centrotemporal IEDs on scalp EEG recordings resulted in a sensitivity of 92.0%, precision of 85.8%, and F1-score of 88.5% in one study^13^, and AUCs of 0.768–0.942 in another^16^. Although it is difficult to directly compare the performance among the models, our good performance for temporal and occipital IED detection might be due to the exclusion of frontal IEDs, which have a low detection performance^12^. In addition, in terms of the source of non-IED epochs, using the non-IED epochs from patients’ EEG recordings resulted in a poorer performance, possibly due to the presence of the abnormal EEG signals in or near the source of the focal IEDs, such as slow activity, voltage attenuation, or alteration of the background synchrony of EEG signals^29,30^.

Another major strength of this study is that it is the first to implement multiclass IED detectors that enable location-specific IED classification. Recent deep learning-based studies have reported multiclass classification for various morphological characteristics of IEDs, such as spikes, sharp waves, broadly distributed sharp waves, and spike-and-wave complexes in scalp EEG recordings^18^; and repetitive high-amplitude complexes, high-amplitude isolated spikes, and atypical epileptiform activities in intracranial EEG recordings^20^. However, multiclass location-specific IED classification could have a clinical advantage over morphology-specific classification as its relevance can be more easily determined and provide clues for epilepsy classification. Considering clinical application of our multiclass IED detectors, we carried out patient-level evaluation for the 2D CNN-based three-class classification which showed the best performance among our multiclass classification approaches in Table 3. It was based on leave-one-patient-out cross-validation which was known to be suitable for the confirmation of model’s generalizability^31^. Our three-class IED detectors provided considerably high detection rates (> 90%) in 57% of the patients with temporal or occipital IEDs, while low (< 66%) in only 13% of the patients, as shown in Table 4. We suggest that our deep learning-based automated multiclass IED detection approaches have a potential for clinical application, provided that we enhance their detection rates for a larger number of patients with a deep understanding of inter-patient variability on electroencephalographic focal IED characteristics. As we concerned that eye-related artifacts could induce unclear interpretations, we did not include frontal IEDs in the patient-level evaluation.

The main reason for the discrepancy in the focal IED detection performance among brain regions in this study might be the different spatial distribution of EEG artifacts and normal EEG variations that might be misinterpreted as IEDs. A review of the major sources of artifacts and their potential implications might help improve the detector performance by annotating those artifacts and normal EEG variations and discriminating them from IEDs in future studies.

Eye-related artifacts are well-known contaminants that can be erroneously interpreted as IEDs in scalp EEG recordings. Eye closure and eye blink result in sharp positive waveforms in the frontal channels (Fp1, Fp2, F3, and F4), while lateral eye movements generate spiky waveforms with high amplitudes in the frontal (Fp1, Fp2, F3, and F4) and anterior temporal (F7 and F8) channels mimicking epileptiform activity if combined with lateral rectus spikes^5,6^. Additionally, eye flutter with myogenic artifacts during photic stimulation can mimic spike-and-wave complexes^32^. Given that eye-related artifacts are predominantly distributed in the frontal region, these may explain the relatively low detection performance of frontal IEDs.

Normal EEG variations are another major cause of erroneous IED interpretation^32–34^. First, fragmented or sharply contoured background alpha rhythm variations could mimic spike-like waveforms in the occipital region, while alpha rhythms in the temporal and occipital regions might have an apiculate morphology^32–35^. The 6–11 Hz wicket waves in the mid-temporal regions are well-known normal variants commonly mistaken as IEDs owing to their sharply contoured morphology^32,36^. A previous study reported that incorrect identification of such wicket waves as epileptiform activity was observed in more than 50% of the patients^36^. These normal EEG variations may have influenced the accuracy of telling focal IEDs from non-IEDs in the temporal and occipital regions, more in the binary classification than in differentiating between temporal and occipital IEDs in multiclass classification.

Misclassification of frontal IEDs in the binary classification tended to be primarily of the false positive type, whereas similar frequencies of the false positive and false negative types were found in the multiclass classification. Misclassification of temporal and occipital IEDs in multiclass classification tended to be of the false negative type. Although understanding the discrepancy between the false positive and false negative type frequencies for frontal IEDs was outside the scope of this study, different binary and multiclass classification schemes might have been its source^18^.

We adopted a CNN architecture with multichannel EEG time series as input data considering the clinical environment that neurologists usually reviewed multichannel EEG time series appeared in the form of channel × time in their monitors to manually detect IEDs. Our EEG channel arrangement was the same as that used by our neurologists when they monitored EEG recordings. In addition, we adopted 1D and 2D CNN architectures to compare their effects on the performance of classification models.

The main difference between our 1D and 2D CNN architectures is that the 1D CNN used kernels for all channels simultaneously, while the 2D CNN used kernels for each channel separately to extract features from the 19-channel EEG time series. Therefore, spatial information associated with EEG features from multiple scalp regions has been better exploited in 2D CNN-based classification. A previous study reported that 2D CNN outperformed 1D CNN in differentiating IED from non-IED binary as it combines temporal and spatial information^10^. From this perspective, we hypothesized that our 2D CNN-based classification models outperformed 1D CNN-based ones unless EEG recordings were severely contaminated by artifacts because kernels of the 2D CNN could extract epileptic electroencephalographic features from each channel more precisely than those of the 1D CNN. However, the performance of the 2D CNN-based classification noticeably declined for frontal IED detection in both binary and four-class classifications, possibly resulting from adverse effects of eye-related artifacts. To explore the difference of the performance between the 1D and 2D CNN-based classification models in more detail, we visualized their corresponding feature maps using t-SNE. We qualitatively examined that the 2D CNN-based classification separated focal IED features more distinctly than the 1D CNN-based one, particularly for three IED classes.

In this study, the frequency of false-positive frontal IEDs in the 2D CNN-based binary classification was higher than that in the 1D CNN-based classification, and the number of false-negative occipital IEDs in the 2D CNN-based multiclass classification was higher than that in the 1D CNN-based classification. In terms of the region-specific misinterpretation of focal IEDs, we suggest that 2D CNN captures the morphological characteristics of eye-related artifacts in frontal regions and normal variations in occipital regions more sensitively than 1D CNN. The number of false-positive frontal IEDs in the 2D CNN-based four-class classification was lower than that in the 1D CNN-based classification, while the number of falsely classified frontal IEDs as temporal and occipital IEDs was higher, indicating a decreased sensitivity in the 2D CNN-based multiclass classifications in detecting frontal IEDs. Although 2D CNN extracts spatial information better than 1D CNN, the additional spatial information probably provoked unintended and mixed region-specific effects on the performance of the classification procedures.

This study had several limitations. First, we limited the focal IEDs to three categories: frontal, temporal, and occipital. Second, the number of annotated IEDs may have been insufficient for generalizing the study results. Third, the patients with temporal IEDs were significantly older than the other subgroups. Fourth, there was no EEG-level clinical validation of focal IED detectors. To address these limitations, we plan studies that will include centrotemporal or generalized IEDs to expand the region-specific detectability of focal IED detectors; and utilize CNNs with more optimal hyperparameters or other deep learning techniques such as LSTM that effectively analyze time-series data or combined CNNs and LSTM^13^. We also plan semi-supervised approaches using clinician-initiated automated detectors to rapidly annotate IEDs and abundantly acquire training datasets; EEG-level evaluation of our IED detectors to improve their clinical applicability; and explainable artificial intelligence-based studies to understand spatiotemporal model interpretability such as occlusion maps^37^ and gradient-weighted class activation mapping^20^. Additionally, studies that will include a larger number of patients, perform group analysis of specific epilepsy syndromes, include complete clinical data, and handle different EEG channel arrangements could also help.

## Conclusions

This study implemented deep learning-based automated focal IED detectors for detecting and localizing frontal, temporal, and occipital focal IEDs based on scalp EEG recordings of patients with epilepsy. Although we believe our detectors performed reasonably well, we still need to resolve the unintended EEG features that lead to region-specific misinterpretations of focal IEDs.

## Supplementary Information

Supplementary Table 1.

## Data Availability

The datasets generated and analyzed during the current study are not publicly available due retrospective design of the study (waiver of informed consent was approved by IRB) but are available from the corresponding author on reasonable request.
