# Supplementary material for: Deep learning-based automated detection and multiclass classification of focal interictal epileptiform discharges in scalp electroencephalograms
Source: Sci Rep. 2023 Apr 25;13:6755. doi: 10.1038/s41598-023-33906-5 (PMC10130023; doi:10.1038/s41598-023-33906-5)
Supplement: Supplementary file 1 — Supplementary Table 1. [file 41598_2023_33906_MOESM1_ESM.docx]

**Supplementary Table.** Model architectures and output shapes (number of filters @ output dimension) of each layer in our one-dimensional (1D) and two-dimensional (2D) convolutional neural networks (CNNs)

|  | **1D CNN** | **2D CNN** |
| --- | --- | --- |
| Input layer | 19×300 | 19×300 |
| Convolution layer 1 | 64@295 | 32@19×295 |
| Max pooling layer 1 | 64@147 | 32@19×147 |
| Convolution layer 2 | 128@142 | 64@19×142 |
| Max pooling layer 2 | 128@71 | 64@19×71 |
| Convolution layer 3 | 64@66 | 32@19×66 |
| Max pooling layer 3 | 64@33 | 32@19×33 |
|  | Flatten layer | |
|  | Hidden layer | |
|  | Output layer | |
